# Supplementary material for: COVID-19 Vaccine Acceptance and Its Determinants among Migrants in Germany—Results of a Cross-Sectional Study
Source: Vaccines (Basel). 2022 Aug 18;10(8):1350. doi: 10.3390/vaccines10081350 (PMC9413826; doi:10.3390/vaccines10081350)
Supplement: Supplementary file 1 [file vaccines-10-01350-s001.zip › vaccines-1819610-supplementary.pdf]

Teil A: A – Experience with COVID-19

At the beginning we would like to know what your experiences are in regard to COVID-19.

A1. Have you ever been infected with COVID-19?

- Yes ☐
- No ☐

A2. If you have been infected with COVID-19: How severe was the infection?

- I was tested positive, but I didn’t experience any symptoms ☐
- I’ve had mild symptoms (like a cold) ☐
- I’ve had severe symptoms (like influenza) ☐
- I was hospitalized (but did not need artificial ventilation) ☐
- I was hospitalized and needed artificial ventilation ☐

A3. Have people in your family or in your circle of friends been infected with COVID-19?

- Yes ☐
- No ☐

Teil B: B – Vaccination status

Now we are going to ask you a few questions about the vaccination against COVID-19.

B1. Are you vaccinated against COVID-19?

- Yes, at least the first dose ☐
- No, but I already have an appointment for the vaccination ☐
- No, and I don’t have an appointment for the vaccination ☐

B2. How strongly do you agree or disagree with the following statements regarding the access to the COVID-19 vaccination?

	1 Strongly agree	2	3	4	5	6	7 strongly disagree	no answer	I don't know
There is a vaccination centre/doctor’s office close to me.	☐	☐	☐	☐	☐	☐	☐	☐	☐
I can reach the vaccination centre/doctor’s office with ease (by car or public transport).	☐	☐	☐	☐	☐	☐	☐	☐	☐
It was easy for me to make an appointment for the vaccination.	☐	☐	☐	☐	☐	☐	☐	☐	☐
I had to wait a long time for an appointment.	☐	☐	☐	☐	☐	☐	☐	☐	☐
Language barriers made it difficult for me to get a vaccination/still make it difficult to get a vaccination.	☐	☐	☐	☐	☐	☐	☐	☐	☐
Paying for the vaccination was/is a problem for me.	☐	☐	☐	☐	☐	☐	☐	☐	☐
My insurance status makes it difficult for me to get the COVID-19 vaccination.	☐	☐	☐	☐	☐	☐	☐	☐	☐

Teil C: C – Willingness to get vaccinated

Next we would like to know what you think about vaccination against COVID-19.

C1. How strongly do you agree or disagree with the following statements regarding the vaccination against COVID-19 (regardless of your own vaccination status)?

	1 Strongly agree	2	3	4	5	6	7 strongly disagree	no answer	I don't know
I think the vaccination against COVID-19 is sensible.	☐	☐	☐	☐	☐	☐	☐	☐	☐
I'm concerned about potential side effects.	☐	☐	☐	☐	☐	☐	☐	☐	☐
I think the COVID-19 vaccine may not be safe.	☐	☐	☐	☐	☐	☐	☐	☐	☐
I don't think that COVID-19 is dangerous to my health.	☐	☐	☐	☐	☐	☐	☐	☐	☐
I am against vaccination in general.	☐	☐	☐	☐	☐	☐	☐	☐	☐
The best way is to let nature take its course.	☐	☐	☐	☐	☐	☐	☐	☐	☐
I believe natural or traditional remedies.	☐	☐	☐	☐	☐	☐	☐	☐	☐
I'm afraid of injections.	☐	☐	☐	☐	☐	☐	☐	☐	☐
I refuse vaccination for religious reasons.	☐	☐	☐	☐	☐	☐	☐	☐	☐

C2. In your opinion, what other reasons speak against a COVID-19 vaccination?

C3. With which of the available vaccines would you want to be vaccinated/have you been vaccinated?

- BioNTech /Pfizer ☐
- Moderna ☐
- AstraZeneca ☐
- Johnson & Johnson ☐
- I don’t want to be vaccinated. ☐
- Other/combination of different vaccines: ☐

Other/combination of different vaccines:

Teil D: D – Attitude towards vaccination

Now we would like to know what you are thinking about vaccination in general.

D1. Please indicate whether you disagree or agree with each of the following statements about childhood vaccine.

	1 Strongly agree	2	3	4	5	6	7 strongly disagree	no answer	I don't know
Vaccines are safe.	☐	☐	☐	☐	☐	☐	☐	☐	☐
Vaccines contain dangerous ingredients.	☐	☐	☐	☐	☐	☐	☐	☐	☐
Some vaccines are unnecessary since they target relatively harmless diseases.	☐	☐	☐	☐	☐	☐	☐	☐	☐
Vaccines are effective at preventing diseases.	☐	☐	☐	☐	☐	☐	☐	☐	☐
In Germany, children are given the right number of vaccines.	☐	☐	☐	☐	☐	☐	☐	☐	☐
In Germany, children are given too many vaccines.	☐	☐	☐	☐	☐	☐	☐	☐	☐
Vaccines conflict with my belief that children should use natural products and avoid toxins.	☐	☐	☐	☐	☐	☐	☐	☐	☐
Vaccines are a major advancement for humanity.	☐	☐	☐	☐	☐	☐	☐	☐	☐
The government should not force children to get vaccinated to attend school.	☐	☐	☐	☐	☐	☐	☐	☐	☐
To protect public health, we should follow government guidelines about vaccines.	☐	☐	☐	☐	☐	☐	☐	☐	☐

D2. Have you been vaccinated in the past five years? (not against COVID-19, but against another disease.)

Yes ☐

No ☐

Teil E: E – Status and sources of information

Now we we would like to know how you found out about the COVID-19 vaccination and how satisfied you are with the information available.

E1. How often do you inform yourself about the COVID-19 vaccination?

- Never ☐
- Partly ☐
- Very often ☐

E2. Where do you get your information about the COVID-19 vaccination? (Multiple answers possible)

- Press, television, radio ☐
- Internet (in general) ☐
- Health authorities (Robert-Koch-Institute, ministry of health, etc.) ☐
- Social media (Facebook, Twitter, Blogs) ☐
- Conversations with family, friends, other acquaintances ☐
- Doctor, pharmacy ☐
- Instant-messenger-service (WhatsApp, Signal, Telegram) ☐
- I don’t inform myself. ☐
- Other: ☐

Other:

E3. How well would you say you feel informed about the COVID-19 vaccination?

- Very good ☐
- Rather good ☐
- Moderate ☐
- Rather bad ☐
- Bad ☐

E4. How would you rate the information on the COVID-19 vaccination?

	Agree	Rather agree	Partly agree/partly disagree	Rather disagree	Disagree
The information supports the decision for or against the vaccination.	☐	☐	☐	☐	☐
The information is easy to assess.	☐	☐	☐	☐	☐
The information is easy to understand.	☐	☐	☐	☐	☐
The information is easy to find.	☐	☐	☐	☐	☐

E5. On which topics would you like to have more information? (Multiple answers possible)

- Duration of the protection after the vaccination ☐
- Vaccine side effects ☐
- Differences between vaccines ☐
- Person to contact in case of vaccine side effects ☐
- Ways to report vaccine side effects ☐
- Benefits of vaccination ☐
- Vaccination appointment ☐
- Time of vaccination ☐
- Necessity of vaccination ☐
- Location where the vaccination takes place ☐
- I have no need for information ☐

Teil F: F – Concept of disease

You are almost done with the survey! The next questions address what you think about COVID-19 and how dangerous you think it is.

F1.

	0 not at all	1	2	3	4	5	6	7	8	9	10 severely
How much does COVID-19 affect your life?	☐	☐	☐	☐	☐	☐	☐	☐	☐	☐	☐

F2.

	0 a short time	1	2	3	4	5	6	7	8	9	10 a long time
How long do you think COVID-19 will continue?	☐	☐	☐	☐	☐	☐	☐	☐	☐	☐	☐

F3.

How much control do you think you have over COVID-19?

0 Absolutely no control	1	2	3	4	5	6	7	8	9	10 Full control
☐	☐	☐	☐	☐	☐	☐	☐	☐	☐	☐

F4.

How much do you think existing treatments help patients with COVID-19?

0 My treatment cannot help me at all	1	2	3	4	5	6	7	8	9	10 My treatment can help me very much
☐	☐	☐	☐	☐	☐	☐	☐	☐	☐	☐

F5.

How much do patients with COVID-19 experience symptoms from the illness?

0 no symptoms at all	1	2	3	4	5	6	7	8	9	10 many severe symptoms
☐	☐	☐	☐	☐	☐	☐	☐	☐	☐	☐

F6.

How concerned are you about COVID-19?

0 not concerned at all	1	2	3	4	5	6	7	8	9	10 extremely concerned
☐	☐	☐	☐	☐	☐	☐	☐	☐	☐	☐

F7.

How well do you feel you understand COVID-19?

0 I do not understand it at all	1	2	3	4	5	6	7	8	9	10 I understand it very good
☐	☐	☐	☐	☐	☐	☐	☐	☐	☐	☐

F8.

How much does COVID-19 affect you emotionally?

0 it does not affect me emotionally at all	1	2	3	4	5	6	7	8	9	10 it extremely affects me emotionally
☐	☐	☐	☐	☐	☐	☐	☐	☐	☐	☐

F9. Please list below the three most important factors that you think are causes of COVID-19:

Teil G: G – Demographics

Finally, we would like to ask you a few more questions about yourself and your living conditions.

G1. What is your gender?

- Male ☐
- Female ☐
- Divers ☐
- No answer ☐

G2. How old are you? (Please enter years)

[ ]

G3. In which country were you born?

- Germany ☐
- Afghanistan ☐
- Iran ☐
- Iraq ☐
- Syria ☐
- Turkey ☐
- Morocco ☐
- Other country: ☐

Other country:

[ ]

G4. If you did not tick “Germany” in question 52: How long have you been living in Germany? (Please enter years)

[ ]

G5. What is your mother tongue/first language?

- German ☐
- Arabic ☐
- English ☐
- Farsi/Dari ☐
- French ☐
- Spanish ☐
- Turkish ☐
- Other language: ☐

Other language:

G6. How many years did you attend school?

- Not at all ☐
- Less than 10 years ☐
- 10 years ☐
- More than 10 years ☐

G7. Where do you live?

- In a rented apartment ☐
- In a condominium ☐
- In my own house ☐
- In a student hostel ☐
- In a shared apartment ☐
- In a community accommodation for refugees ☐

G8. What is your residence status?

- German citizenship ☐
- Permanent residence permit (Unbefristete Aufenthaltsgenehmigung) ☐
- Temporary residence permit (Befristete Aufenthaltsgenehmigung) ☐
- ongoing asylum procedure (Aufenthaltsgestattung) ☐
- temporary suspension of teportation (Duldung) ☐
- Other: ☐

Other:

____

G9.

	very good	good	moderate	bad	very bad
How would you describe your current general state of health? It is…	☐	☐	☐	☐	☐

Thank you for your participation in the survey!

If you want to take part in the raffle, please click on the link below.
